# Endometriosis and epithelial ovarian cancer: a two-sample Mendelian randomization analysis

**DOI:** 10.1038/s41598-023-49276-x

**Published:** 2023-12-11

**Authors:** Li Wang, Xuri Li, Yan Wang, Guofeng Li, Shuzhen Dai, Mengying Cao, Zhen Meng, Songtao Ren

**Affiliations:** 1 Department of Gynecology & Obstetrics, Liaocheng People’s Hospital, School of Medicine, Liaocheng University, Liaocheng, China; 2 Department of Gynecology, Qingdao Traditional Chinese Medicine Hospital, Qingdao Hiser Hospital Affiliated of Qingdao University, Qingdao, China; 3 Department of Medical Record Management, Fourth People’s Hospital of Liaocheng, Liaocheng, China; 4 Department of Physical Treatment, Fourth People’s Hospital of Liaocheng, Liaocheng, China; 5 Department of Gynecology, The Affiliated Hospital of Qingdao University, Qingdao, China; 6 Biomedical Laboratory, School of Medicine, Liaocheng University, Liaocheng, China; 7 Department of Neurosurgery, Liaocheng People’s Hospital, Liaocheng, China

**Keywords:** Cancer, Risk factors

## Abstract

Endometriosis, a prevalent condition, has long been recognized as a chronic and debilitating ailment affecting an estimated 1790 million women worldwide. Observational studies have established a correlation between endometriosis and ovarian cancer. Thus, we endeavored to employ Two-Sample Mendelian Randomization, utilizing summary statistics from a Genome-Wide Association Study of endometriosis and epithelial ovarian cancer, with genetic markers serving as proxies for epithelial ovarian cancer. The analysis revealed a significant correlation between these entities, with an odds ratio (OR) of 1.23 (95% CI 1.11–1.36). Upon histotype-specific examination, robust evidence emerged for an association of endometriosis with the risk of endometrioid carcinoma (OR 1.49, 95% CI 1.24–1.81), clear cell carcinoma (OR 2.56, 95% CI 1.75–3.73), and low malignant potential tumors (OR 1.28, 95% CI 1.08–1.53). These findings provide a theoretical framework for prospective investigations aimed at enhancing the potential therapeutic efficacy of managing endometriosis in averting the onset and progression of ovarian cancer.

## Introduction

Endometriosis, recognized as a prevalent ailment, stands as a chronic, incapacitating condition^1,2^, affecting 5–10% of women of reproductive age worldwide^3^. The interconnection between endometriosis and malignancy has been subject to extensive scrutiny over several years. Substantial data accrual substantiates the notion that certain gynecological cancers may arise from endometriosis, a supposition reinforced over the past few decades^4^. Ovarian cancer which is the most lethal gynecological malignancy^5^ and second most common cause of gynecologic cancer death in women around the world^6^ is often associated with endometriosis, especially the relatively uncommon carcinoma of the ovary clear cell carcinoma^4^. Risk factors associated with epithelial ovarian cancer encompass a family history of this malignancy, the cumulative count of lifetime ovulations, as well as benign gynecological conditions, notably endometriosis^7^, and potentially use of talcum powder^8^. Indeed, endometriosis has been reported to be associated with a heightened risk of several types of cancer in population-based research^9^. Sampson's pioneering report in 1927 alluded to the malignancy associated with endometriosis, wherein he delineated specific criteria for endometriosis-associated ovarian cancers^10^. The meta-analyses of relationships between endometriosis and ovarian cancer have been published these years. The meta-analysis including 24 observational studies to evaluate the association between endometriosis and ovarian cancer calculated summary relative risk 1.93 (SRR = 1.93, 95% CI 1.68–2.22) of ovarian cancer among women with endometriosis compared with those without^11^. Another study, based on 21 case–control or cohort studies published between 1990 and 2012, estimated the summary relative risk (SRR) of endometriosis on ovarian cancer to be 1.27 (95% CI = 1.21–1.32), and 1.80 (95% CI = 1.28–2.53) based on five studies exclusively involving women with endometriosis^12^. Wang et al.^13^ reported an odds ratio (OR) of 1.42 (95% CI = 1.28–1.57) based on 12 case–control studies.

Accurately quantifying the risk of ovarian cancer in women afflicted with endometriosis holds paramount significance for various reasons. Nevertheless, these findings present intricate management dilemmas for clinicians tending to women affected by endometriosis and may carry substantial public health ramifications^14^. This is especially true in the realms of cancer screening and prevention for women, as well as in the enduring care strategies implemented by healthcare practitioners for women grappling with endometriosis^15^.

While there exists a discernible association between endometriosis and epithelial ovarian cancer, establishing a causal link remains equivocal. Mendelian randomization (MR) emerges as a pivotal tool to illuminate this intricate interplay^16^. This analytical research approach furnishes substantial insights into putative causal relationships between modifiable risk factors and diseases^17^. It leverages genetic variation as a natural experiment, enabling the investigation of causal connections between potentially modifiable risk factors and health outcomes within observational datasets^16,18–20^. MR, in comparison to conventional observational studies, is less susceptible to the confounding effects or reverse causation, thereby conferring significantly augmented statistical power in two-sample inquiries^17^. It is imperative to acknowledge, however, that these advantages are contingent upon two additional assumptions: firstly, that the two samples inherently represent the same underlying population, and secondly, that any overlap in participants between the two samples may introduce bias in the risk factor-outcome association^21,22^. Two-Sample MR analysis is a statistical method employed MR to infer causal relationships between exposures and outcomes. It leverages genetic variants associated with the exposure of interest as instrumental variables, capitalizing on their random allocation during conception. This approach helps mitigate issues related to confounding and reverse causation, common in observational studies.

In light of the ambiguous causal relationship between previously observed associations of endometriosis and the development of epithelial ovarian cancer, this study undertook a two-sample MR analysis utilizing published (Genome-Wide Association Study) GWAS data. The aim was to scrutinize the connection between endometriosis and various facets of epithelial ovarian cancer, including its histotypes, as well as low malignant potential tumors.

## Methods

### Study design

Two-sample MR analysis^23^ was used to estimate the casual relationship between endometriosis and the risk of ovarian cancer. This Two-sample MR study is an extension based on MR that is a form of instrumental variable analysis that uses genetic variants to proxy for environmental exposures^24^ and the effects of the genetic instrument on the exposure and on the outcome in the Two-sample MR are obtained from separate GWAS.

### Identification of SNPs associated with endometrioses

For Endometriosis, we used the GWAS (discovery and replication meta-analysis, including 17,045 Endometriosis cases and 191,596 controls.)^25^ which identified and extract information for single nucleotide polymorphisms (SNPs) that were associated with Endometriosis at the genome-wide significance level (*P* < 5 × 10^8^).

### GWAS of ovarian cancer

To assess whether Endometriosis is associated with ovarian cancer, we used data from a GWAS of epithelial ovarian cancer (EOC) using DNA samples from OncoArray Consortium consisted of 25,509 women with epithelial ovarian cancer and 40,941 controls of European ancestry that passed Quality Control (QC)^26,27^. This database comprised 63 genotyping project/case–control sets representing participants recruited from 14 countries. The analyses included 66,450 samples from 7 genotyping projects: 40,941 controls, 22,406 invasive cases including 1012 low-grade serous, 13,037 high-grade serous, 2810 endometrioid, 1366 clear cell, 1417 mucinous and other 2764 EOC. Analyses were also performed for 3103 borderline cases including 1954 serous borderline and 1149 mucinous borderline tumors. Genotypes for OCAC samples were preferentially selected from the different projects in the following order: OncoArray, Mayo GWAS, Collaborative Oncological Gene-environment Study (COGS), and other GWAS. SNP QC was carried out according to standard QC guidelines^26^ and datas were obtained either by direct genotyping using an Illumina Custom Infinium array (OncoArray) considering of approximately 530,000 SNPs or by imputation with reference to the 1000 Genomes reference panel phase 3 version 5^28^. Ethical approval from relevant research ethics committees was granted for each of the original GWAS studies and details can be found in the respective publications.

### Statistical analyses

Genetic instruments for exposures used in an MR framework allows for unbiased causal effects of risk factors on disease outcomes to be estimated is based on three key assumptions: (i) genetic instrument is robustly associated with the risk factor of interest; (ii) the instrument must not be associated with any confounding factor(s) of the association between the exposure and outcome; and (iii) there must be no effects of the genetic variants on the outcome, that do not go via the risk factor (i.e. no horizontal pleiotropy)^29^. SNPs were pruned for linkage disequilibrium at R^2^ < 0.001 at a clumping distance of 10,000 kilobases from the lead SNP at *P* < 5 × 10^−8^ with reference to the 1000 Genomes Project (https://www.internationalgenome.org) when obtaining effect estimates from relevant GWAS.

We conducted Two-sample MR analyses using an inverse variance weighted (IVW) to estimate the association between endometriosis and ovarian cancer. IVW is a weighted linear regression model, which aggregates and minimizes the sum of the variances of two or more random variables, and each random variable is inversely proportional to its variance^30^.

We identified 19 SNPs associated with Endometriosis from GWAS publication, after screening for correlation and removing linkage disequilibrium, a final set of 14 SNPs was obtained, and these 14 SNPs were included in our instrument (rs10167914; rs10757272; rs11674184; rs12037376; rs12700667; rs1448792; rs1537377; rs17803970; rs1903068; rs1971256; rs2206949; rs6546324; rs71575922; rs74485684). 14 SNPs were used to construct the genetic instrument for endometriosis. The primary MR analysis was conducted using the IVW method, wherein the SNP to outcome estimate is regressed on the SNP to exposure estimate, all genetic variations are valid instrumental variables (IV)^29^. Fixed effects IVW were used to give that we did not detect instrument variable assumption violations (neither heterogeneity nor pleiotropy were observed). MR-Egger regression^29^, weighted median estimation^31^, and weighted mode estimation^32^, each of which makes different assumptions about the underlying nature of horizontal pleiotropy were also built. Additionally, leave-one-out permutation analyses were performed to examine whether any results were driven by individual influential SNPs in IVW models^33^.The proportion of risk factor and outcome variance could explained by SNPs used as instruments in this method, to help establish whether SNPs associated with both risk factors and outcomes, further to primarily represent (1) a direct association of a SNP with a risk factor, which then influences an outcome, or (2) a direct association of a SNP with an outcome, which then influences the level of a risk factor^33^.We conducted Cochran’s *Q* and Rucker’s *Q* heterogeneity tests for IV using IVW and MR-Egger methods^23,34^.

ORs [95% confidence intervals (CI)] were estimated in all invasive ovarian cancers, borderline disease and by histotype (serous borderline, mucinous borderline, low-grade serous, high-grade serous, mucinous invasive, clear cell and endometrioid) samples. MR Egger regression to assess bias from directional pleiotropy^26^, and using a weighted median estimator that can provide a consistent estimate of the effect when ≤ 50% of the information comes from invalid instrumental variables^31^. All analyses were conducted in R studio and R (version 3.6.3) with the packages (‘TwoSampleMR’^18,35^ and MendilianRandomization).

### Ethics approval

Ethical approval from relevant research ethics committees was granted for each of the original GWAS studies and details can be found in the respective publications.

## Results

In the IVW models, compelling evidence indicated a significant association between persistent endometriosis and the risk of epithelial ovarian cancer (OR per year: 1.23, 95% CI 1.11–1.36; *P* = 5.44E−05). Additionally, we assessed the association of the 14-SNP instrument with endometriosis using MR-Egger, weighted median, and weighted mode techniques. MR-Egger regression showed an OR of 1.72 (95% CI 1.11–2.66), while both weighted median (OR 1.26, 95% CI 1.13–1.41) and weighted mode (1.29, 95% CI 1.10–1.52) techniques provided directionally consistent results. These findings suggest that the results remain robust even in the face of potential violations of MR assumptions. Furthermore, the intercept value of 0.99 (OR 0.96–1.01, *P* = 0.34) in MR-Egger regression indicates no statistical significance. This implies that genetic pleiotropy did not influence the MR results (Table 1). In the context of heterogeneity testing, the results of the Cochran’s* Q* test and Rucker’s* Q* test are *P* = 0.133 and *P* = 0.169 respectively, indicating no heterogeneity among the IV.

**Table 1:** IVW and sensitivity analysis estimates for the association of Endometriosis with risk of Ovarian Cancer.

Exposure	Outcome	Method	OR	95% CI	*P*-value
Endometriosis	Ovarian Cancer	IVW	1.23	1.11–1.36	5.44E−05
		MR-Egger regression	1.72	1.11–2.66	0.032
		Weighted median	1.26	1.13–1.41	3.42E−05
		Weighted mode	1.29	1.10–1.52	0.01

**Figure 1:**
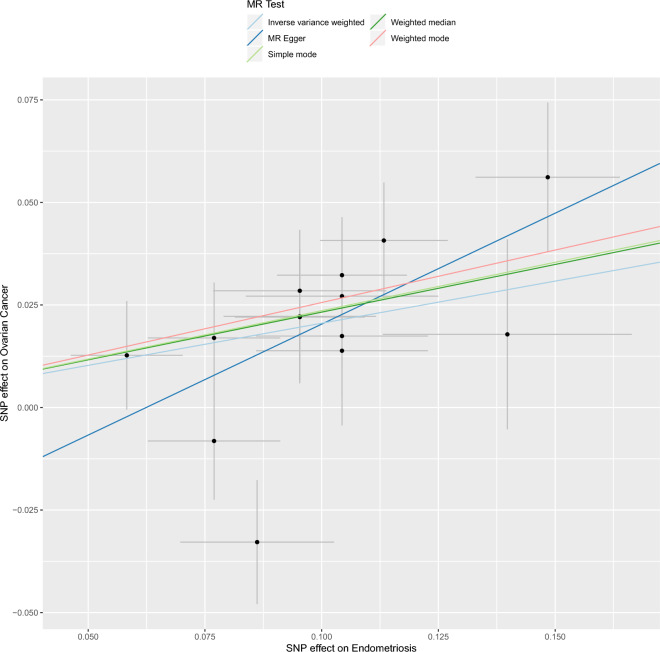
Scatter plot of single SNP with Endometriosis as the exposure and Ovarian Cancer as the outcome.

**Figure 2:**
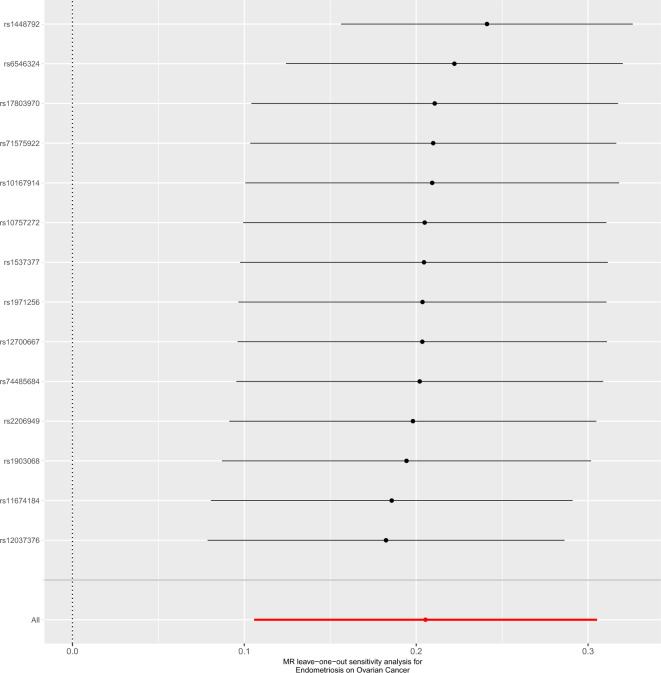
Leave-one-out analysis of single SNP with Endometriosis as the exposure and Ovarian Cancer as the outcome.

**Figure 3:**
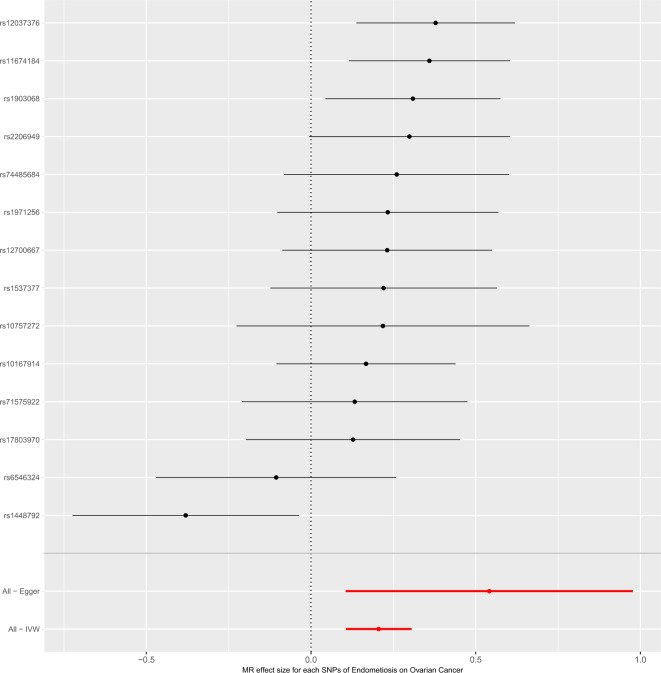
Forest plot analysis of single SNP with Endometriosis as the exposure and Ovarian Cancer as the outcome.

To obtain MR estimates for each individual SNP, we conducted the analysis multiple times for each exposure-outcome combination. In each iteration, a different single SNP was used for the analysis. We also assessed the association of the 14-SNP instrument with endometriosis, as demonstrated in Table 2. The scatterplots depicted in Fig. 1 illustrate that SNPs exerting a larger effect on endometriosis also exhibit a greater impact on the risk of ovarian cancer. Each method is represented by a different colored line, with the slope of the line indicating the estimated causal effect. It is feasible to conduct a leave-one-out analysis, wherein the MR analysis is repeated while excluding each SNP individually. This allows us to discern that a majority of the associated signals were not primarily influenced by a single genetic marker, as demonstrated in the leave-one-out analysis presented in Fig. 2. We employed a forest plot to juxtapose the MR estimates derived from various MR methods with those obtained from single SNP tests. This plot illustrates the estimates of endometriosis when assessed with each individual SNP, juxtaposed against the causal effect estimated using methods that incorporate all the SNPs (Fig. 3).

**Table 2:** Association analysis of the SNPs instrument with Endometriosis with the diagnosis of Ovarian Cancer.

Exposure	Outcome	SNPs	OR	95% CI	*P*-value
Endometriosis	Ovarian cancer	rs10167914	1.182	0.90–1.55	0.23
		rs10757272	1.24	0.80–1.94	0.34
		rs11674184	1.43	1.12–1.83	0.004
		rs12037376	1.46	1.15–1.86	0.002
		rs12700667	1.26	0.92–1.73	0.15
		rs1448792	0.68	0.48–0.96	0.03
		rs1537377	1.25	0.88–1.96	0.21
		rs17803970	1.14	0.82–1.57	0.44
		rs1903068	1.36	1.04–1.78	0.02
		rs1971256	1.26	0.90–1.77	0.17
		rs2206949	1.35	0.99–1.83	0.055
		rs6546324	0.90	0.62–1.29	0.57
		rs71575922	1.14	0.81–1.61	0.45
		rs74485684	1.30	0.92–1.83	0.14

For histotype-specific analyses, there was suggestive evidence for an association of endometriosis with risk of endometrioid carcinoma (OR 1.49, 95% CI 1.24–1.81, *P* = 3.18E−05), clear cell carcinoma (OR 2.56, 95% CI 1.75–3.73, *P* = 1.09E−06) and low malignant potential tumors (OR 1.28, 95% CI 1.08–1.53, *P* = 0.005) in IVW models showed in Table 3, which was consistent in sensitivity analyses examining horizontal pleiotropy. Moreover, we conducted the analysis multiple times for each exposure-outcome combination, utilizing a different single SNP each time for the analysis. This process involved analyzing 14 SNPs as instruments for endometriosis, considering the three histotype-specific outcomes as described previously (Table 4).

**Table 3:** IVW and sensitivity analysis estimates for the association of Endometriosis with risk of invasive epithelial ovarian cancer histotypes and low malignant potential tumors.

Outcome Ovarian Cancer	IVW OR (95% CI)	*P*-value	MR-Egger regression OR (95% CI)	*P*-value	Weighted median OR (95%C)	*P*-value	Weighted mode OR (95% CI)	*P*-value
HGSC	1.10 (0.99–1.23)	0.09	1.68 (1.06–2.67)	0.05	1.11 (0.96–1.27)	0.15	1.13 (0.87–1.46)	0.36
LGSC	1.07 (0.79–1.44)	0.66	0.90 (0.23–3.53)	0.88	1.06 (0.70–1.60)	0.77	1.00 (0.54–1.87)	1.00
Mucinous	1.29 (0.98–1.68)	0.07	1.48 (0.41–5.33)	0.56	1.07 (0.75–1.52)	0.71	0.93 (0.49–1.76)	0.82
Endometrioid*	1.49 (1.24–1.81)	3.18E−05	1.28 (0.52–3.16)	0.60	1.48 (1.16–1.90)	0.002	1.46 (1.02–20.9)	0.06
Clear cell*	2.56 (1.75–3.73)	1.09E−06	7.69 (1.41–41.78)	0.054	2.94 (2.03–4.26)	1.32E−08	3.00 (1.79–5.04)	0.001
LMP*	1.28 (1.08–1.53)	0.005	2.37 (1.06–5.30)	0.057	1.27 (1.01–1.61)	0.04	1.33 (0.92–1.94)	0.15

Causal estimates are scaled to represent the association of endometriosis persistent exposure.

*IVW* Inverse-variance weighted, *HGSC* High grade serous carcinoma, *LGSC* Low grade serous carcinoma, *LMP* Low malignant potential tumours.

*Suggestive evidence for an association of endometriosis with risk of diagnosis.

**Table 4:** Association analysis of the single SNPs instrument with Endometriosis with the histotypes of Ovarian Cancer.

SNPs	Endometrioid Carcinoma			Clear cell Carcinoma			Low malignant potential tumour			Associated Gene/cytoband
	OR	95% CI	*P*-value	OR	95% CI	*P*-value	OR	95% CI	*P*-value	
rs10167914	1.30	0.72–2.34	0.39	4.51	2.02–10.06	0.00	1.48	0.83–2.64	0.19	Interleukin (IL)-1A/2q13
rs10757272	1.92	0.73–5.01	0.18	2.83	0.74–10.85	0.13	0.65	0.25–1.68	0.38	CDKN2BAS/9p21.3
rs11674184	1.51	0.89–2.57	0.13	3.08	1.46–6.47	0.003	1.26	0.75–2.13	0.38	GREB1/2p25.1
rs12037376	1.47	0.87–2.46	0.15	2.20	1.09–4.46	0.02	1.89	1.11–3.14	0.01	WNT4/1p36.12
rs12700667	1.53	0.77–3.05	0.23	3.51	1.32–9.33	0.01	1.24	0.63–2.44	0.53	Inter-genic region upstream of plausible candidate genes NFE2L3 and HOXA 10./7p15.2
rs1448792	0.82	0.39–1.72	0.59	0.32	0.11–0.91	0.033	0.92	0.44–1.92	0.83	LINC01239 RNA/9p21.3
rs1537377	1.45	0.69–3.05	0.33	1.65	0.58–4.66	0.35	1.27	0.61–2.65	0.52	CDKN2B-AS1/9p21.3
rs17803970	1.02	0.51–2.06	0.96	5.08	1.78–14.50	0.002	0.97	0.49–1.94	0.93	SYNE1/6q25.1
rs1903068	1.52	0.85–2.70	0.16	4.09	1.81–9.27	0.001	1.58	0.90–2.79	0.11	KDR/4q12
rs1971256	3.26	1.60–6.63	0.001	3.01	1.11–8.15	0.03	0.76	0.37–1.57	0.46	CCDC170/6q25.1
rs2206949	1.95	1.01–3.75	0.05	2.64	1.06–6.55	0.04	1.69	0.89–3.22	0.11	ESR1/6q25.1
rs6546324	1.18	0.53–2.60	0.68	0.72	0.24–2.21	0.57	1.35	0.62–2.94	0.45	ETAA1/2p14
rs71575922	0.88	0.42–1.86	0.75	6.01	2.21–16.33	0.00	1.14	0.56–2.39	0.71	SYNE1/6q25.1
rs74485684	2.93	1.38–6.23	0.005	1.76	0.61–5.02	0.29	1.30	0.62–2.69	0.49	FSHB/11p14.1
IVW	1.50	1.24–1.81	3.18E−05	2.56	1.75–3.73	1.09E−06	1.28	1.08–1.53	0.005	
MR-Egger	1.28	0.52–3.15	0.60	7.69	1.41–41.78	0.05	2.37	1.06–5.30	0.06	

## Discussion

This study employed Two-sample MR analyses, leveraging extensive data from large-scale GWAS of the OCAC, to scrutinize the interplay between endometriosis and epithelial ovarian cancer. Our findings indicate a causal relationship, revealing that heightened endometriosis persistence corresponds to an elevated risk of ovarian cancer. Furthermore, we provide detailed results regarding the association with specific histotypes of epithelial ovarian cancer, including low malignant potential tumors.

Endometriosis affects approximately 10% of the female population, and its impact extends beyond adverse effects on quality of life and infertility. Emerging data suggest that considering the potential for malignant transformation is crucial^36^. Among associated cancers, ovarian cancer takes precedence, ranking as the second most common cause of gynecologic cancer-related mortality worldwide^6^. Notably, the primary subtypes linked to endometriosis are endometrioid and clear cell carcinoma^36^.

Several observational studies have explored the link between endometriosis and ovarian cancer. Nonetheless, it is important to note that there is compelling evidence indicating a potential publication bias, which may lead to an overestimation of this association^11^. The correlation between genetic predisposition to endometriosis and the development of invasive epithelial ovarian cancer supports previous findings from traditional analyses, affirming that women with endometriosis face an increased risk of developing this form of cancer^13,37^. A comprehensive MR analysis^33^ utilizing 10 selected SNPs as instrumental variables, yielded compelling evidence for a link between genetic susceptibility to endometriosis and the occurrence of invasive epithelial ovarian cancer (OR per 50% higher odds liability to endometriosis: 1.10, 95% CI 1.06–1.15; *P* = 6.94 × 10^–7^). However, in our Two-sample MR analysis, we calculated the consistent results that is women with endometriosis has 23% (OR = 1.23 95% CI 1.11–1.36; *P* = 5.44E−05) greater risk of epithelial ovarian cancer using 14 SNPs as instruments, which was consistent in different models and across sensitivity analyses examining horizontal pleiotropy. As MR studies can provide reliable evidence on the effect of modifiable endometriosis as risk factor for ovarian cancer as outcome and can overcome some limitations of traditional observational epidemiology^17^. We could definite the endometriosis as the causal or etiology of epithelial ovarian cancer but not only a risk factor.

The associations we observed between endometriosis and epithelial ovarian cancer risk by histotype using Two-sample MR, lend support to the previous observational study results. In histotype-specific analyses, there was suggestive evidence for an association of endometriosis with risk of endometrioid carcinoma (OR 1.49, 95% CI 1.24–1.81, *P* = 3.18E−05), clear cell carcinoma (OR 2.56, 95% CI 1.75–3.73, *P* = 1.09E−06) and low malignant potential tumors (OR 1.28, 95% CI 1.08–1.53, *P* = 0.005), which was consistent in sensitivity analyses examining horizontal pleiotropy. Although endometriosis is reported to be present in 25–58% of clear cell carcinoma cases and be considered as a risk factor for its developing^10,38–41^, most of them concerned the association between clear cell carcinoma and endometriosis^42,43^, which is risk of clear cell carcinoma was found significantly elevated in the patients with endometrioma of the ovary (RR/relative risk = 12.4)^42^. Few studies have produced risk estimates endometriosis and histotype of ovarian cancer and Saavalainen^44^ found that endometrioma was positively associated with the clear cell (SIR = 10.1), endometrioid (SIR = 4.7) and serous (SIR = 1.62) histotypes. A pooled analysis of 13 case–control studies including 7911 women with ovarian cancer and 13,226 controls showed that self-reported endometriosis was associated with an increased risk of ovarian clear cell carcinoma (OR = 3.05, 95% CI 2.43–3.84), endometrioid carcinoma (OR = 2.04, 95% CI 1.67–2.48), and low grade serous carcinoma (OR = 2.11, 95% CI 1.39–3.20)]^45^, reinforcing that the higher risk of ovarian cancer in women with endometriosis is restricted to the clear cell, endometrioid and low malignant ovarian cancer histotypes, and future studies should focus on these three histotypes rather than on ovarian cancer overall. The clear cell OR from IVW was smaller than MR regression, but the effect estimates and confidence intervals obtained from MR-Egger regression, WM, and WME are consistent with those from IVW. All four methods yield significant p-values, and the direction of causal effects obtained from the four algorithms aligns. Furthermore, we found different SNPs associated with different histotype, also suggests that subclinical manifestations of or pathways leading to oncogenesis, indicating important avenues for future mechanistic work. While we detected little association between endometriosis and serous or mucinous cancer, for HGSC (OR = 1.10 95% CI 0.99–1.23, *P* = 0.09), LGSC (OR = 1.07 95% CI 0.79–1.44, *P* = 0.66) and Mucinous (OR = 1.29 95% CI 0.98–1.68, *P* = 0.07).However, ovarian clear cell carcinoma is a different entity from the other endometriosis-associated ovarian carcinomas with a distinct gene expression profile^46^.

Although observational and MR estimates examining associations between endometriosis and risk of epithelial ovarian cancer are qualitatively similar, it is important to emphasize that observational effect estimates for disease states examined cannot be compared quantitatively to the MR estimates in our study. In settings for which the instrumental variable assumptions are well justified (assessed as described above and using biological knowledge), the findings could help prioritise clinical trials or drug development and inform clinical or public health decision making^22,47,48^.

Additionally, further work to understand the possible mechanisms through which factors that appear to influence epithelial ovarian cancer in endometriosis promote oncogenesis could help to increase the scope for prevention opportunities across the life course. The mechanisms governing the varying associations between endometriosis and specific types of ovarian cancer require in-depth exploration. It is imperative to uncover fundamental discoveries pertaining to omic-driven pathways associated with the pathophysiologic patterns specific to endometriosis-related cancer. This endeavor will shed light on why women with endometriosis may face an elevated risk of certain forms of epithelial ovarian cancer. The well-being of women with endometriosis may be influenced by care decisions that could stem from potential misinterpretations of the connection between endometriosis and ovarian cancer^49^. Furthermore, the long-term management of women with endometriosis should pay more concern on the screening of malignant diseases.

Our study features a rigorous application of two-sample MR, utilizing genetic variants as instrumental variables. We integrated comprehensive data from GWAS of both endometriosis and epithelial ovarian cancer. This robust methodology, coupled with extensive data sources, bolsters our causal inference by mitigating biases stemming from confounding and reverse causation. Furthermore, our findings provide a solid theoretical framework for future investigations focused on refining therapeutic approaches for endometriosis. This potential refinement holds promise in reducing the risk and progression of ovarian cancer, carrying substantial implications for clinical management.

There are several limitations in our research. Our research also has some limitations. First, the Two-sample MR analysis used summarized data from GWAS catalog and it was not possible to detect the nonlinear relationship between exposure factors and disease outcomes. Second, the Two-sample analysis cannot explain the pathogenesis of epithelia ovarian cancer, so it is necessary to further explore the pathogenic mechanism of endometriosis on epithelial ovarian cancer, especially for the clear cell, endometrioid and low malignant histotypes. Finally, these findings need to be carried out in the context of other evidence with specific associations, but the MR method we used in this study can provide strong support for clarifying the direction of causality. In this way, we believe that this study can contribute to clinical treatment for endometriosis and prevention efforts of epithelial ovarian cancer to improve public health.

## Conclusion

In conclusion, our results furnish compelling evidence of a causal link between endometriosis and heightened risk of epithelial ovarian cancer, particularly notable in clear cell, endometrioid, and low malignant potential histotypes. These findings establish a theoretical framework for future investigations, aiming to enhance the efficacy of endometriosis life management in averting the onset and progression of ovarian cancer. Subsequent studies are warranted to delve into the underlying mechanistic pathways governing this association.

## Data Availability

The datasets generated and analyzed during the current study are available in the ieu open gwas project repository, https://gwas.mrcieu.ac.uk/.
